# 1994. Infections following Chimeric Antigen Receptor (CAR) – T cell Therapy: 2018-2022

**DOI:** 10.1093/ofid/ofad500.121

**Published:** 2023-11-27

**Authors:** Vishakh C Keri, Jahanavi M Ramakrishna, Rahul Vyas, Lea M Monday, Abhinav Deol, Pranatharthi Chandrasekar, Mahmoud Al-Saadi

**Affiliations:** Wayne State University, Detroit, Michigan; Wayne State University, Detroit, Michigan; Wayne State University, Detroit, Michigan; Wayne state University School of Medicine, Detroit, Michigan; Karmanos Cancer Institute, Detroit, Michigan; Wayne State University, Detroit, Michigan; Wayne State University, Detroit, Michigan

## Abstract

**Background:**

Chimeric Antigen Receptor (CAR) T-cell therapy is an emerging therapeutic modality for relapsing and refractory haematological malignancies. We describe the infectious complications following CAR T-cell therapy.

**Methods:**

This is a retrospective analysis of data on patients who received CAR-T cell therapy between April 2018 and December 2022 at Karmanos Cancer Institute, Detroit, Michigan. Patients’ data were collected up to their last known clinic or inpatient follow-up visit. An infectious episode was defined as any microbiologically proven or documented infection. Statistical analysis was performed using SPSS software version 28.0.

**Results:**

Seventy-six patients received therapy with FDA-approved CAR-T cell products. Thirty-three patients (43.4%) had at least one infection with a range of 1 to 4 infectious episodes per patient. There were 61 infectious episodes during a median follow-up of 184 (96-340) days. The median duration for the onset of infection was 59 (22-209) days. Bacterial and viral infections occurred in 42.6% and 41% of the infectious episodes respectively. COVID-19 was the most common infectious complication (14.8%). Time-to-event analysis showed that most of the infections occurred within the first 100 days (Figure 2). Empirical antibiotic use in Cytokine Release Syndrome (CRS)/ Immune effector Cell-Associated Neurotoxicity Syndrome (ICANS) in the absence of documented infection was reported in 85.7% of patients. *Clostridioides difficile (C.difficile*) accounted for 11.5% of all infections. Out of 6 patients with *C.difficile* infection 5 patients had CRS and antibiotic use. There were four documented deaths during the study period. Two deaths occurred in patients who had an infection.

**Figure:**
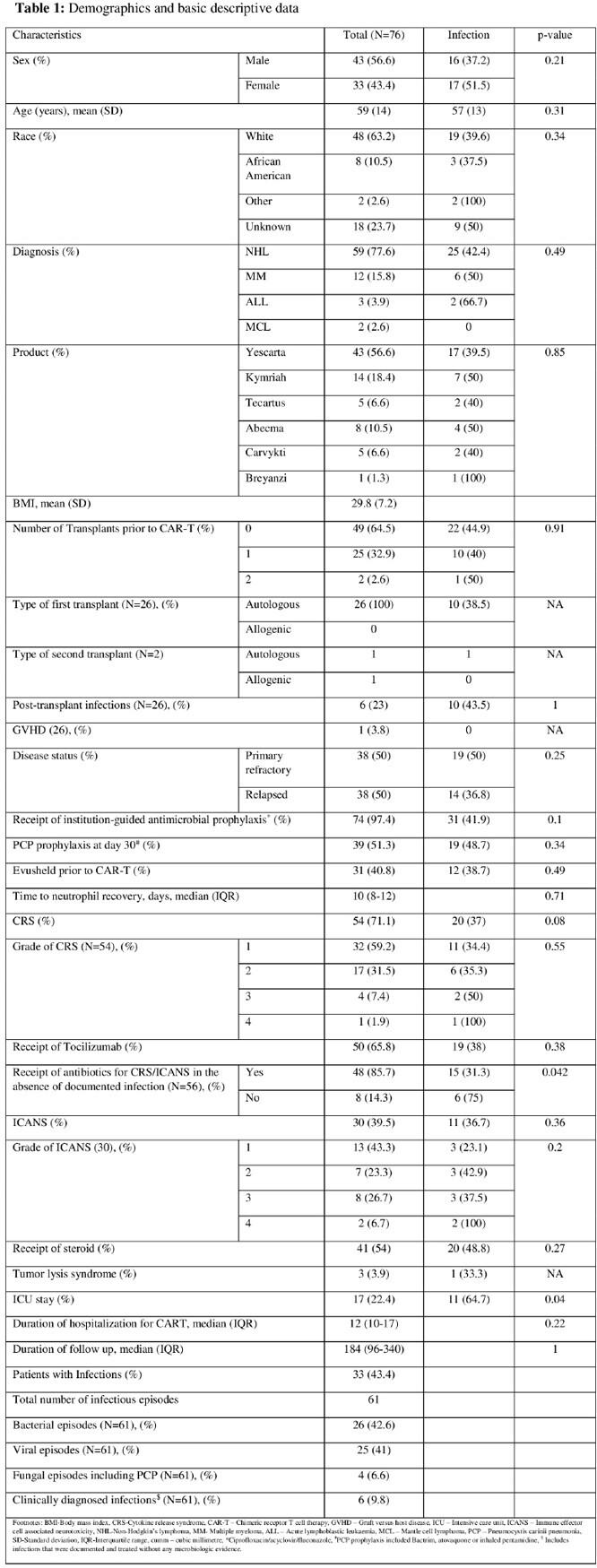

**Figure:**
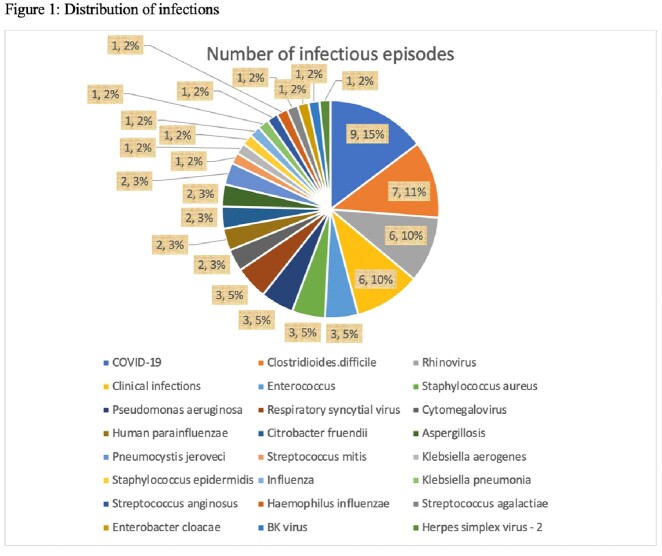

**Figure:**
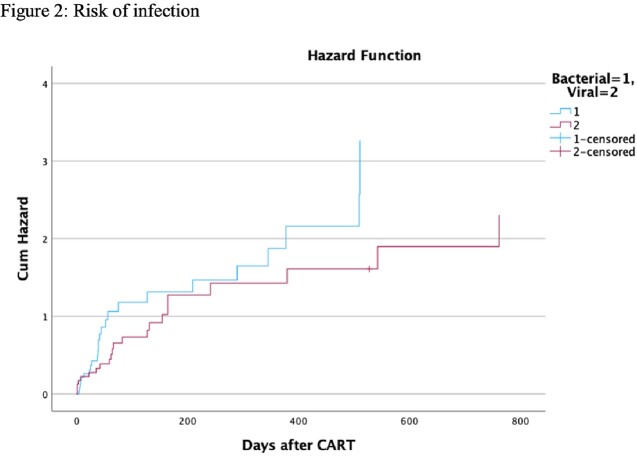

**Conclusion:**

The mortality rate among patients who had an infection was 6.5%. Most of the infections occurred within the first 100 days. COVID-19 and *C. difficile* infection were the most common infections following CAR-T cell therapy. The rate of *C. difficile* infection was high in patients with CRS receiving empiric antibiotics in the absence of any documented infection, thus providing an opportunity for antibiotic stewardship in this population.

**Disclosures:**

**All Authors**: No reported disclosures

